# Case of Hæmatoid Tumor of the Brain

**Published:** 1825-12

**Authors:** George Gregory

**Affiliations:** senior Physician of the St. George's and St. James's Dispensary, &c.


ART. III.—*Case of Hæmatoid Tumor of the Brain.* By GEORGE GREGORY, M.D. senior Physician of the St. George's and St. James's Dispensary, &c.

GEORGE LANCASHIRE, residing at No. 5, Queen street, Grosvenor-square, being employed in the repair of a house, had the misfortune to fall from a twelve-step ladder, and received a severe blow upon his head. This happened in June, 1823, when he was sixteen years of age. He was stunned by the fall, and was soon after visited by a medical practitioner, who did not, however, think it necessary to bleed him. Six weeks after the accident, he had a severe epileptic fit; after which he was bled and blistered, by which means he so far recovered as to be enabled to resume his usual employments. But, though he continued at his work, he was comparatively feeble, and he made constant complaints of headache. On the 8th of May, 1825, being then at Brighton, he had a second epileptic fit, of great severity, which left him so weak that it was the 5th of June before he was able to remove to London. From that period he never left his bed-room. A third long and severe fit occurred on the 15th of June; and several, of nearly equal violence, followed during the succeeding months. He was completely confined to his bed towards the end of August, and lingered miserably till the 17th of October, when death put a period to his sufferings.

He came under my care as a patient of the St. George's and St. James's Dispensary, on the 17th September. He was then sufficiently sensible to describe to me the excruciating pain he had long suffered in the fore part of the head. His mother informed me that, for hours together, she had sat pressing his forehead with both her hands, as the only means of allaying the torture he experienced. When I first visited him, a relaxation of the bowels had come on, and he passed his stools and urine involuntarily. The headache had diminished in violence; the pupils were much dilated; the pulse was 120, sharp; the tongue clean. His body was greatly emaciated; his weakness such, that he could with difficulty be raised to have his bed made. I ordered him an astringent mixture.

On the 13th and 14th of October, he had a succession of epileptic fits. The stomach now became so irritable, that nothing was retained. The tongue was morbidly red; the pulse but little affected. An epileptic fit carried him off early in the morning of the 17th October.

His body was opened the following day by Mr. Richard Bushel, an intelligent pupil of the Dispensary, in the presence of myself, Dr. Milligan, Mr. Lightfoot, of Oxford-street, (who had attended the patient at a former period of his illness,) and several students. A note of the appearances on dissection was kindly furnished by Mr. Bushel; aud it is so comprehensive and accurate, that I have much pleasure in submitting it to the readers of your valuable and widely-read Journal.

---

*Sectio Cadaveris (thirty-three hours after Death).*

Upon the removal of the calvarium, (which was observed to be somewhat thinner than usual,) the surface of the dura mater did not present any morbid appearance. This membrane having been divided and turned back, the substance of the encephalon looked of a pale yellow colour, and was of a softer texture in parts than natural; the superficies of the anterior lobe of the left hemisphere was smooth and uniform, the convolutions and intergyral spaces being nearly effaced. The upper part of the cerebrum being removed on a level with the corpus callosum, and an opening made into the right lateral ventricle, some bloody serum escaped, and a considerable quantity of crassamentum (apparently of recent effusion, and the immediate cause of death,) remained filling the whole of its cornua.

The left ventricle was afterwards examined, and found full of serum, with some portion of coagulum in its inferior cornu, that being the most depending part. This had made its way from the opposite side, the pressure there having caused a laceration of the septum lucidum, or partition of the ventricles.

Having divided the corpus callosum, and tracing from whence the blood could proceed that was seen in the ventricles, it was found to communicate with a cyst formed in the substance of the left anterior lobe, also filled with coagulated blood, which adhered rather firmly to its internal surface. The parietes of this cavity or cyst were distinct, and could be peeled from the medullary substance, which, in the upper part of the lobe corresponding to the smooth surface, was nearly absorbed.

In the anterior cornu of the left ventricle, and in front of the corpus striatum, a tumor, the full size of a nutmeg, of a hard and fleshy consistence, was observed. A section was then made through this, when it presented a laminated structure. This body, with the cyst which adhered to it, were removed, and afterwards immersed in alcohol, for the purpose of more minute inspection. The parts after some time becoming indurated, another incision was made, which opened into a small cavity in its centre. The divided surfaces were nearly an inch in thickness, and similar to those of coagulated blood, when treated in a like manner.

The cerebellum, the upper part of the medulla oblongata, and the basis of the brain, were examined; but no diseased appearances were there met with.

The thoracic, as well as the abdominal viscera, upon investigation, were found perfectly healthy.

